# Relationship between Food Security, Nutrition Security, and Diabetes: The Role of Supplemental Nutrition Assistance Program Participation

**DOI:** 10.1016/j.cdnut.2024.102153

**Published:** 2024-03-30

**Authors:** Maha Almohamad, Jayna M Dave, Eric E Calloway, Ruosha Li, Shreela Sharma

**Affiliations:** 1 Department of Epidemiology, Human Genetics and Environmental Sciences, The University of Texas Health Science Center at Houston (UTHealth) School of Public Health, Houston, TX, United States; 2 US Department of Agriculture/ Agricultural Research Service Children’s Nutrition Research Center, Department of Pediatrics, Baylor College of Medicine, Houston, TX, United States; 3 Gretchen Swanson Center for Nutrition, Omaha, NE, United States; 4 Department of Biostatistics and Data Science, The University of Texas Health Science Center at Houston (UTHealth) School of Public Health, Houston, TX, United States

**Keywords:** nutrition insecurity, food insecurity, SNAP, diabetes, moderation analysis

## Abstract

**Background:**

Inadequate nutrition and poor diet quality are associated with a heightened risk of diabetes. The connection between food insecurity measures and diabetes has been established, with evidence indicating that Supplemental Nutrition Assistance Program (SNAP) participation contributes to reductions in food insecurity. Recently developed nutrition security measures, defined as the ability to acquire healthful foods to prevent diseases, and their association with diabetes and SNAP participation are not yet understood.

**Objectives:**

This study aimed to assess the relationship between food security and nutrition security in relation to diabetes overall and by SNAP participation and nutrition security as potential modifiers.

**Methods:**

Secondary data analysis of cross-sectional pilot study data collected from adults in 5 US states (N = 517). Logistic regression mixed models included moderation analysis and clustering effects by state to address site-level confounding.

**Results:**

Higher nutrition security scores among adults, after adjusting for confounders, were significantly associated with lower odds of diabetes risk (adjusted odds ratio = 0.59; 95% confidence interval: 0.40, 0.87; *P* value = 0.008). Statistically significant interaction effect of differences according to SNAP participation was observed for nutrition security (*P*_homogeneity/interaction_ = 0.021), adjusting for age, gender, race/ethnicity, education, employment, National School Lunch Program, Special Supplemental Nutrition Program for Women, Infants, and Children, food pantry use, household with children, survey mode, and food security. The association between food security and diabetes was not statistically significant overall. However, statistically significant interaction effect of differences according to SNAP participation was observed for food security (*P*_homogeneity/interaction_ = 0.047). Further, no interaction effect of differences in nutrition security was found between food security and self-reported diabetes/prediabetes (*P*_homogeneity/interaction_ = 0.250).

**Conclusions:**

This study sheds light on the early exploration of the intricate relationship between nutrition security and diabetes. The findings suggest that a higher nutrition security score, after adjusting for confounders, was significantly associated with lower odds of diabetes risk. Notably, there were statistically significant interaction effects in these associations based on SNAP participation.

## Introduction

Food insecurity, defined as the state of being without reliable access to sufficient quantities of affordable foods, remains a critical issue in the United States (US) [1]. In 2019, prior to the COVID-19 pandemic, ∼13.7 million (10.5%) US households were categorized as food insecure [2], and high rates of food insecurity persisted even after the pandemic [2]. Millions of US adults have been at risk of extreme poverty, with many undernourished people continuing to rise with no means to provide nutritious foods for themselves and their families in 2020 [3]. In 2021, 10.2% of US households were affected by food insecurity [1].

Research shows that individuals living in food insecure households reported higher risks of developing chronic diseases, including diabetes [[4], [5], [6]]. The prevalence of diabetes among individuals living in food insecure households is significantly higher compared with individuals living in food secure households, even after controlling for confounders [7,8]. Food insecurity has been associated with poor glycemic control among adults with diabetes [9].

Food security, which includes 4 dimensions (availability, accessibility, utilization, and stability), is a well-established social determinant of health linked to disease-related health outcomes [10]. However, food security does not inherently address diet quality or nutrition assessments [11]. This highlights the need to explore nutrition security, which is distinct from food security, defined as “consistent and equitable access to healthy, safe, affordable foods essential to optimal health and well-being” [12]. To further support our point, the Food and Agriculture Organization of the United Nations (FAO) and the US Department of Agriculture (USDA) recognize that nutrition is a critical aspect of human well-being and sustainable food systems [13,14].

Nutrition assistance programs such as the Supplemental Nutrition Assistance Program (SNAP) are effective in reducing food insecurity [[15], [16], [17]]. SNAP focuses on households with low income and households at or <130% of the poverty line, and among those eligible, helps to ensure benefits are adequate to afford a nutritious diet in an average month to support healthy eating patterns [11,18]. Increasing SNAP benefits among those eligible may facilitate dietary improvements and help reduce food insecurity among those with diabetes [19]. SNAP is a highly effective program shown to reduce food insecurity by 30% [20,21]. Nonetheless, SNAP primarily focuses on food assistance and does not address nutrition for disease prevention and nutrition security [22].

Currently, addressing food insecurity in the US has somewhat eliminated caloric insufficiencies but has not fully addressed obesity and undernourishment, often linked to poor diet quality [23]. Nutrition security is related to food security; however, it is a novel, emerging concept that is distinct from food security [11]. Nutrition security is a construct that builds on the food security construct, emphasizing the role of nutrition in health. It will coexist with food insecurity and diet-related disease and disparities [18]. Further, nutrition security includes secure access to nutritiously adequate foods, health services, and care [24]. Although conjectured, food insecurity measurements alone could underestimate the actual effect of the negative relationship between food insecurity and diabetes, in which the relationship could be attenuated.

A better understanding of nutrition security and its association with diabetes is needed to understand whether a relationship exists, considering the potential modifying role of SNAP. The COVID-19 pandemic raised awareness of the disrupted food systems and increased food insecurity, which led to policies highlighting the quantity of food over the quality of food [25], creating a higher need to further research this area [26]. Given the recent development and validation of a nutrition security measure in the US by the Gretchen Swanson Center for Nutrition (GSCN) [27], this study offers a timely assessment of the relationships among these variables.

The purpose of our study is to assess the relationship between nutrition security and food security as independent predictors of diabetes. Additionally, the moderating effect of participation in nutrition assistance program SNAP on the relationship between nutrition security, food security, and diabetes was explored. Finally, the moderating effect of nutrition security on the relationship between food security and diabetes was also explored.

## Methods

### Study design

This cross-sectional study employed a secondary data analysis approach using existing data to address a new research question. The secondary analysis of data was derived from a pilot study conducted by GSCN, which focused on newly developed and validated measures that provide a more holistic assessment of the experiences related to food security and nutrition security (N = 517) [27].

### Study population

The study sample included low-income adults residing in California, Florida, Maryland, North Carolina, or Washington. Inclusion criteria included age ≥18 y old, understanding of English, ability to answer questions about themselves and the household, and being from a household experiencing food insecurity or at risk of food insecurity assessed by the 18-item Household Food Security Survey Module (HFSSM) by the USDA [27,28].

### Data source

Data were obtained from GSCN [27], which created a survey including items for the new measures, scales, and items that assess food insecurity constructs and demographic questions [27]. In addition to food and nutrition security, other social determinants of health were examined, in line with prior research on structural disparities. Secondary analysis of data from a pilot study was collected from ∼500 households that were predominantly low-income and/or food insecure [27]. Participant recruitment was site-based; participants were recruited from food pantries, Federally Qualified Health Centers (FQHCs), and other programs that serve populations at risk of food insecurity. Participants were recruited via email, text messages, and/or flyers. One person from each household completed the survey and received a $25 gift card for completing it. The survey was ∼75–85 questions depending on skip patterns, delivered in English only, with ∼71% web-based surveys and 29% paper surveys. Representation from multiple regions of the US was optimal; however, due to capacity limitations, the study was limited to 5 states. Sample size was predetermined based on available data, collected from April to June 2021 [27].

### Definition and measurement of outcome, exposures, and covariates

#### Self-reported health outcome

Items were assessed from the Centers for Disease Control and Prevention’s (CDC) Behavioral Risk Factor Surveillance System survey [29]. The dependent variable (outcome) was self-reported diabetes/prediabetes coded as a binary variable (1 = yes, 0 = no). The survey questions grouped diabetes/prediabetes risk.

#### Household food security exposure

The USDA HFSSM [30], 18-item version was used to assess household food insecurity [28]. Using a 12-mo recall period, the sum score of affirmative responses to the 18-item USDA Household Food Security Survey scale was between 0 and 10 without children and between 0 and 18, with children with higher scores indicating a higher degree of food insecurity. Households were assigned food security categories based on the number of affirmative (i.e., “Sometimes true” or “Often true”) responses (0 affirmative responses = “High food security”; 1–2 affirmative responses = “Marginal food security”; 3–7 for households with children or 3–5 for households without children = “Low food security”; 8–18 for households with children or 6–10 for household without children = “Very low food security”) [28]. These categories were treated as a 4-level ordinal variable for the analyses, scored from 0 = “High food security” to 3 = “Very low food security.” For the analysis in this article, the exposure was food security status using this categorical scoring [food secure (reference level), marginal food security, low food security, very low food security].

#### Household nutrition security exposure

Household nutrition security data were collected using newly developed measures and questionnaires by GSCN, which encompassed a broader range of factors beyond those captured by the HFSSM [27]. Rather than focusing on food access, nutrition security measures include a more comprehensive evaluation of nutritional intake. Items were pilot-tested and psychometrically tested [27]. Nutrition security status was assessed by a mean score ranging from 0 to 4, with a higher score indicating a higher degree of nutrition security, in which the household feels free from external constraints and worries about being able to access healthful foods [27]. Response options for all items were five-point ordinal scales from “Always” (Scored as 0) to “Never” (Scored as 4). The items asked included the following: 1) “In the last 12 months, (I/we) had to eat some foods that were not good for my health and well-being because (I/we) couldn’t get other types of food.” 2) “In the last 12 months, (I/we) knew there were things (I/we) should or should not eat for (my/our) health and well-being, but could not get healthful food.” 3) “In the last 12 months, (I/we) worried that the food (I was/we were) able to eat would hurt (my/our) health and well-being.” 4) “In the last 12 months, (I/we) had to eat the same thing for several days in a row because (I/we) didn’t have money to buy other food” [27]. Nutrition security was assessed as an exposure in one model and as a moderator in a separate model.

#### Confounders

**FIGURE 1:**
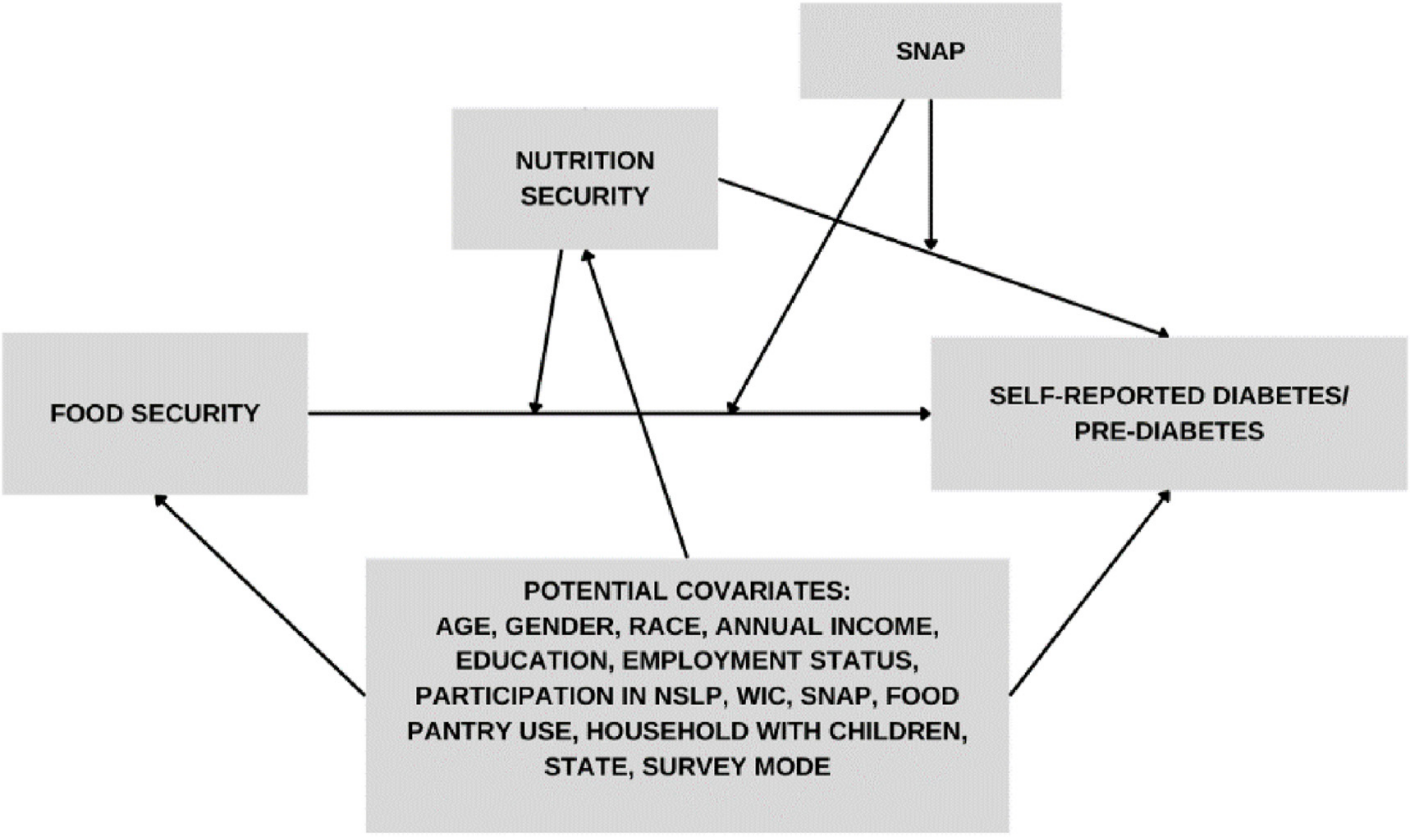
Directed acyclic graph depicting hypothesized relationships between food security, nutrition security, SNAP, and self-reported diabetes/prediabetes. SNAP, Supplemental Nutrition Assistance Program.

Potential confounders were chosen primarily based on the directed acyclic graph (Figure 1) and established literature. Confounders include age (continuous variable in years), gender (binary: 1 = male, 2 = female), race (categorical: 1 = White Non-Hispanic, 2 = Latino/Hispanic, 3 = Black Non-Hispanic, 4 = Multiracial/ethnic or another not listed, Asian Non-Hispanic, or Tribal/Indigenous Non-Hispanic, annual income (continuous- ranging from $3000–$63,000), education (categorical: 0 = less than high school, 1 = high school diploma or general educational diploma, 2 = some college, 3 = Associates degree or greater), employment (categorical: 0 = not working, retired, disabled, a full-time homemaker/stay-at-home parent, or a full-time student, 1 = work in temporary or seasonal job or work year-round <30 h/wk, 2 = work year-round in a job for ≥30 h/wk), participation in the National School Lunch Program (NSLP; free and reduced-price lunch or breakfast program), Special Supplemental Nutrition Program for Women, Infants, and Children (WIC), SNAP, and food pantry use. These nutrition assistance program variables and food pantry use were coded as 0 = no, 1 = yes, and assessed over the past year of current participation. If participants were ineligible for these nutrition assistance programs or did not participate, they were coded as 0 = no for not participating, regardless of eligibility. Other important confounders considered were households with total children (0 = none, 1 = yes), state, and survey mode (online vs. paper-based) [27].

### Descriptive statistics/analyses

#### Descriptive statistics

Frequency distributions of key population demographics were examined by diabetes/prediabetes. Pearson’s Chi-square test was used to examine the association between categorical variables, and a Student’s T-test or Mann-Whitney U test was used to compare the means of two independent groups or continuous variables. These tests assessed statistically significant relationships between variables.

#### Analyses

Univariable analysis was conducted to assess the unadjusted models. Confounder selection relied on prior knowledge supported by a directed acyclic graph, purposeful variable selection with any variable with a significant univariable test (*P* < 0.05) selected as a candidate in the model, and regression adjustment for final confounder selection in the models. Annual income showed no difference between groups in bivariate comparisons and no association with the outcome, so it was excluded from the final models. Based on known biological plausibility, it is sensible to include all other variables as confounders. For the outcome of diabetes/prediabetes, mixed logistic regression analysis was conducted for the two exposures: 1) food security and 2) nutrition security. Overall models adjusted for gender, race/ethnicity, age, education, employment, NSLP, WIC, SNAP, food pantry use, number of children in the household, survey mode, and food security through multivariable mixed effect logistic regression. All mixed models include clustering effects by states to address site-level effects. Food security and nutrition security were assessed as exposures in separate models to avoid multicollinearity. These variables were analyzed separately to assess their individual effects on the outcome to provide a more comprehensive understanding of influencing factors. The relationship between nutrition and food security is shown using an analysis of variance test and boxplot graph (Supplemental Figure 1).

In addition, we examined whether the association was moderated by SNAP by including an interaction term between the exposure and SNAP and adjusting for the selected confounders. Subsequent subgroup analysis was conducted by SNAP [31].

Further, we investigated whether nutrition security moderated the relationship between food security and diabetes. If *P*-interaction/heterogeneity was significant and subgroup-specific estimates differed from one another, there would be a significant interaction effect.

All models relied on case wise deletion to handle missing data. Significance was established at *P* ≤ 0.05. All analyses were conducted using STATA 16.1 statistical software (StataCorp LLC).

## Results

A total of 517 adults aged 18–64 years (35.8% reported very low food security, 26.9% reported low food security, and 13.2% reported marginal food security; 16.1% reported food security) (Table 1) were included in the full analytic sample (Supplemental Figure 2). Among these participants, 23.7% (n = 115) reported having diabetes or prediabetes, with a total mean nutrition security score of 2.6. This sample consisted of 70% females, 21% Hispanic/Latino, 40% White Non-Hispanic, 16% Black Non-Hispanic; 32% had only a high school diploma or General Education Diploma (GED). The study sample comprised a high proportion of individuals who participated in nutrition assistance programs: 33.9% utilized NSLP, 14.7% used WIC, 54.4% participated in SNAP, and 71.6% used food pantries. Surveys were mostly completed online (67.1%) compared with paper-based (26.9%) (Table 1). The relationship between nutrition and food security was statistically significant (*P* value <0.001), indicating higher nutrition security correlated with higher food security, whereas lower nutrition security correlated with lower food security (Supplemental Figure 1).

**TABLE 1:** Sample characteristics of adults who completed the Gretchen Swanson Center for Nutrition pilot survey from April to June 2021

Exposures/Confounders	Diabetes or prediabetes						
	Overall (n = 517)		No (n, %) (n = 371, 71.8)		Yes (n, %) (n = 115, 23.7)		T-test/Chi-square
	n	Mean (SD)	n	Mean (SD)	n	Mean (SD)	*P* value
Nutrition security	432	2.6 (0.88)	325	2.7 (0.86)	107	2.4 (0.89)	0.004∗
Food security	n	%	n	%	n	%	0.000∗
Very low food security	185	35.8	135	36.4	50	43.5	
Low food security	139	26.9	105	28.3	34	29.6	
Marginal food security	68	13.2	59	15.9	9	7.8	
Food secure	83	16.1	65	17.5	18	15.7	
Demographics	n	%	n	%	n	%	*P* value
Gender (%)	486						0.000∗
Male	111	21.5	90	24.3	21	18.3	
Female	364	70.4	271	73.1	93	80.9	
Race/ethnicity (%)							0.000∗
White non-Hispanic	207	40.0	164	44.2	43	37.4	
Latino/Hispanic	109	21.1	72	19.4	37	32.2	
Black non-Hispanic	85	16.4	67	18.1	18	15.7	
Asian non-Hispanic, Tribal/indigenous Non-Hispanic, Multiracial/ethnic or another not listed	66	12.8	50	13.5	16	13.9	
Education (%)							0.000∗
Less than high school	47	9.1	29	7.8	18	15.7	
High school Diploma or GED	165	31.9	133	35.9	32	27.8	
Some college	119	23.0	93	25.1	26	22.6	
Associate degree or greater	134	25.9	97	26.2	37	32.2	
Employment Status (%)							0.000∗
Not working, retired, disabled, a full-time homemaker/stay-at-home parent, or a full-time student	302	58.4	219	59.0	83	72.2	
Work in a temporary or seasonal job or work year-round <30 h/w	88	17.0	71	19.1	17	14.8	
Work year-round in a job for ≥30 h/wk	81	15.7	69	18.6	12	10.4	
	n	Mean (SD)	n	Mean (SD)	n	Mean (SD)	*P* value
Age (years)	486	45.1 (14.6)	371	44.0 (14.5)	115	48.7 (14.4)	0.003∗
Annual income	475	15,890.5 (11,505.4)	361	15,793.6 (12,050.9)	114	16,197.4 (9616.5)	0.744
Nutrition assistance Programs (% yes)	n	%	n	%	n	%	*P* value
NSLP	175	33.9	133	35.9	42	36.5	0.000∗
WIC	76	14.7	55	14.8	21	18.3	0.000∗
SNAP	281	54.4	204	55.0	77	67.0	0.000∗
Food pantry use	370	71.6	281	75.7	89	77.4	0.000∗
Other variables of interest							*P* value
Household with children – Total							0.000∗
None	199	38.5	152	41.0	47	40.9	
Yes	287	55.5	219	59.0	68	59.1	
State							0.000∗
California	117	22.6	75	20.2	42	36.5	
Florida	99	19.2	81	21.8	18	15.7	
Maryland	80	15.5	57	15.4	23	20.0	
North Carolina	95	18.4	80	21.6	15	13.0	
Washington	95	18.4	78	21.0	17	14.8	
Survey mode							0.000∗
Online	347	67.1	259	69.8	88	76.5	
Paper	139	26.9	112	30.2	27	23.5	

Abbreviations: NSLP, National School Lunch Program; SNAP, Supplemental Nutrition Assistance Program; WIC, Special Supplemental Nutrition Program for Women, Infants, and Children.

Missing data for diabetes/prediabetes (n = 31), nutrition security (n = 85), food security (n = 42), gender (n = 42), race/ethnicity (n = 50), education (n = 52), employment status (n = 46), age (n = 31), annual income (n = 42), NSLP (n = 31), WIC (n = 31), SNAP (n = 31), food pantry use (n = 31), household with children (n = 31), state (n = 31), survey mode (n = 31).

∗*P* value <0.05.

Table 2 reports the adjusted odds ratio (AOR) for the association between food security or nutrition security status and self-reported diabetes/prediabetes using separate models. Overall, food insecurity levels were not significantly associated with diabetes compared with food security, adjusting for age, gender, race/ethnicity, education, employment, NSLP, WIC, SNAP, food pantry use, household with children, and survey mode. The effect of food security on diabetes for SNAP participants and nonparticipants was significantly different based on the statistically significant interaction (*P*_homogeneity/interaction_ = 0.047). The fully adjusted model showed a significant positive association with diabetes and very low food security compared with food secure counterparts among nonparticipating SNAP individuals (AOR = 6.11; 95% CI: 1.42, 26.33; *P* value = 0.015) (Table 2). The association between food insecurity and diabetes was nonsignificant among those who participated in SNAP. Further, the effect of food security on self-reported diabetes/prediabetes was not moderated by nutrition security (*P*_homogeneity/interaction_ = 0.250) in the fully adjusted model, and no further stratification was assessed due to insignificance (Table 2).

**TABLE 2:** AORs (95% CI) for association between diabetes/prediabetes and food security or nutrition security, overall and in subgroups by SNAP

Exposure	Subgroup	AOR	95% CI	*P* value	*P*_homogeneity/ interaction_
Food security^1^	Overall (n = 441)^2^				
	Marginal food security	0.57	0.22, 1.47	0.247	
	Low food security	0.99	0.47, 2.11	0.989	
	Very low food security	1.62	0.79, 3.35	0.189	
	By SNAP^3^				0.047∗^4^
	No (n = 181)				
	Marginal food security	0.74	0.10, 5.39	0.769	
	Low food security	1.85	0.40, 8.50	0.432	
	Very low food security	6.11	1.42, 26.33	0.015	
	Yes (n = 260)				
	Marginal food security	0.39	0.12, 1.27	0.118	
	Low food security	0.65	0.25, 1.71	0.386	
	Very low food security	0.77	0.31, 1.96	0.588	
	By nutrition security^2^				0.250^5^
Nutrition security	Overall (n = 399)^6^	0.59	0.40, 0.87	0.008	
	By SNAP^7^				0.021∗^4^
	No (n = 162)	0.59	0.27, 1.29	0.185	
	Yes (n = 237)	0.61	0.37, 0.99	0.049	

Abbreviations: AOR, adjusted odds ratio; CI, confidence interval; NSLP, National School Lunch Program; *P*_*homogeneity/interaction*_, *P* value of the interaction term in the multivariable logistic regression model; SNAP, Supplemental Nutrition Assistance Program; WIC, Special Supplemental Nutrition Program for Women, Infants, and Children.

∗*P* value <0.05.

1 Reference = food secure.

2 Fully adjusted logistic regression models included age, gender, race/ethnicity, education status, employment status, NSLP, WIC, SNAP, food pantry use, household with children, survey mode; Random effect by state.

3 Fully adjusted logistic regression models included age, gender, race/ethnicity, education status, employment status, NSLP, WIC, food pantry use, household with children, survey mode; Random effect by state.

4 Effect Modification was calculated by SNAP.

5 Effect Modification was calculated by nutrition security.

6 Fully adjusted logistic regression models included age, gender, race/ethnicity, education status, employment status, NSLP, WIC, SNAP, food pantry use, household with children, survey mode, food security; Random effect by state.

7 Fully adjusted logistic regression models included age, gender, race/ethnicity, education status, employment status, NSLP, WIC, food pantry use, household with children, survey mode, food security; Random effect by state.

Households with a higher mean score for nutrition security indicated higher nutrition security. Nutrition security was inversely associated with diabetes, such that a unit increase in household nutrition security score was associated with 41% lower odds of having diabetes (AOR = 0.59; 95% CI: 0.40, 0.87; *P* value = 0.008), adjusting for age, gender, race/ethnicity, education, employment, NSLP, WIC, SNAP, food pantry use, household with children, survey mode, and food security status (Table 2). Statistically significant interaction effect according to SNAP participation was observed for nutrition insecurity (*P*_homogeneity/interaction_ = 0.021), indicating the association varied depending on SNAP participation. The fully adjusted model showed a significant inverse association between diabetes and nutrition security among participating SNAP individuals, indicating a potentially protective effect of nutrition security against diabetes among SNAP participants. Among SNAP participants, higher nutrition security scores were associated with 39% lower odds of diabetes/prediabetes (AOR = 0.61; 95% CI: 0.37, 0.99; *P* value = 0.049) compared with those not participating in SNAP (Table 2). The association was nonsignificant among individuals who participated in SNAP.

## Discussion

This is one of the first studies to assess nutrition security and its association with diabetes. Nutrition security was assessed using a new set of measures developed and validated by GSCN [27]. This study found that adults with higher nutrition security and who participated in SNAP had lower odds of self-reported diabetes/prediabetes compared with those with lower nutrition security and who did not participate in SNAP. Participation in SNAP had a moderating effect on this relationship, i.e., it strengthened the association between nutrition security and diabetes. Conversely, this study also found that food security was not statistically associated with diabetes, even after adjusting for confounders. Findings suggested that very low food security was associated with significantly higher odds of self-reported diabetes/prediabetes among nonparticipating SNAP individuals, highlighting the importance of food security in this population. Moreover, the interaction between food security and nutrition security was not significant, suggesting that whereas both are important factors to consider, their combined effect on diabetes/prediabetes is not significantly different from their individual effects.

This study stands out as an early investigation into the relationship between nutrition security and diabetes, with no existing literature available for comparison of the findings. By understanding the potential effects of nutrition security, assessment of the populations most in need of support can be enhanced. This knowledge will help future stakeholders strategize interventions to connect these populations with food services and resources, thereby mitigating the risk of diabetes. It is important to note that individuals can experience food insecurity with nutrition insecurity (e.g., low quantity and low quality of food) or food insecurity without nutrition insecurity (e.g., low quantity of food but not low quality). Moderation analysis was conducted to explore the underlying mechanisms of the observed association between food security and diabetes.

Prior studies have demonstrated a significant relationship between food insecurity and diabetes prevalence [10,[32], [33], [34]]. Other studies have demonstrated relationships between food insecurity and self-reported diabetes [6]. Moreover, the research on food insecurity and diabetes has been inconclusive, with some studies finding no significant association between food insecurity and diabetes among a large sample of adults in the US [35]. The current study extends the research by reporting a statistically significant relationship between food security and diabetes when considering modification by SNAP participation. However, nutrition security was significantly associated with diabetes. There is little to no past research assessing the association between nutrition security, specifically, and diabetes in the US. The CDC’s National Center for Chronic Disease Prevention and Health Promotion and the American Heart Association advocate for improving food and nutrition security to achieve health equity through successful initiatives [26].

To address these food insecurity issues, nutrition assistance programs such as SNAP were developed [31]; however, overall cardiovascular disease (CVD) risk factors such as diabetes remain unchanged. Previous studies showed the prevalence of obesity and diabetes did not decline among SNAP participants [36]. SNAP, the largest federal nutrition assistance program, strives to improve the dietary intake of participants who experience food insecurity by providing food items and nutrition education [16]. Among households receiving SNAP benefits, >50% experienced food insecurity [37]. The relationship between food insecurity and SNAP has been established, with SNAP participation shown to reduce food insecurity [15]. Meanwhile, more studies examining the impact of nutrition assistance programs on cardiovascular health have been recommended to address the gaps in understanding the association between food insecurity and CVD [37]. One impediment has been the food insecurity screening tools, which do not include diet quality or nutrition assessments [22]. For example, the 2 most commonly used assessment tools, the 2-item Hunger Vital Scale and the 18-question household food security assessment by the USDA, do not include questions about nutrition and diet quality [22,38,39].

Effectively addressing diet-related diseases requires a shift to this refined concept of nutrition security [26], and innovative approaches are needed to address important CVD risk factors that food insecurity alone may not be sufficient to explain.

### Limitations and strengths

This study has a few limitations. First, a cross-sectional study design limits the ability to make strong causal inferences. Food security and nutrition security were measured at the household level, introducing misclassification bias at the individual level. The counts among categories of food security were small. The self-reported survey is based on a 12-month recall period, which could introduce recall bias. Also, using self-reported diabetes/prediabetes as the outcome may be inaccurate and subject to recall bias. The convenience sample used in this study may not represent households with food insecurity in the US. Moreover, measurement error could have been introduced due to the ability to incorrectly follow skip patterns with paper surveys, whereas they cannot do it in an online survey. Males were not well represented in this study as the sample mostly comprised females. Several unexpected null findings were shown in this study as well, which may be related to the sample size. However, these results are an important early investigation into the relationship between newly developed nutrition security measures and diabetes. Lastly, results should be interpreted with caution because even though we were able to detect some differences, the study may be underpowered to detect some of these associations, particularly in the SNAP subgroups. The strengths of this study included a paper and online survey mode, which reduces sampling error and allows for a better representation of the target population of those who do and do not have access to the internet. Further, this is the first assessment of this topic in the US with a fairly large and diverse sample.

### Implications and conclusion

There is a gap in our knowledge regarding the relationships between food and nutrition security, and diabetes/prediabetes. This study provides opportunities to derive further hypotheses to understand the longitudinal risk of food and nutrition security with diabetes risk and develop interventions and policies to mitigate these risk factors. Further, this study offers valuable insights into the impact of nutrition security on the association between food security and diabetes. These findings can assist policymakers in tailoring programs like SNAP to better address this relationship. A long-term goal should be to establish a standardized conceptualization of nutrition security. Such work could inform intervention approaches to support those who experience nutrition insecurity and promote collaboration between community-based organizations, food banks and pantries, policymakers, and healthcare providers to ensure the availability and accessibility of nutritious foods for good health and well-being. Addressing and improving nutrition security could lead to lower risk of adverse CVD risk factors such as diabetes/prediabetes. The study contributes to a more comprehensive understanding of the interplay between food access, nutrition quality, and health outcomes, ultimately informing strategies to address food-related health disparities in the US.

## Author contributions

The authors’ responsibilities were as follows – MA, JD, and SS designed research; MA conducted research; EC provided essential materials; MA and RL analyzed data; MA wrote the article; JD, SS, RL, and EC critically reviewed the article; MA had primary responsibility for final content; and all authors: read and approved the final manuscript.

## Conflicts of interest

The authors report no conflicts of interest.

## Funding

This work is a publication of the USDA/ARS Children’s Nutrition Research Center, Department of Pediatrics, Baylor College of Medicine, Houston, TX, USA. This project was partly supported through federal funds from the US Department of Agriculture (USDA)/Agricultural Research Service under Cooperative Agreement No. 3092-51000-058-2S (JD). The contents of this publication do not necessarily reflect the views or policies of the USDA, nor does mention of trade names, commercial products, or organizations imply endorsement by the US Government. This work was supported by the Center for Health Equity at UTHealth Houston School of Public Health. Gretchen Swanson Center for Nutrition, with funding from the Walmart Foundation, supported publication funding.

### Data availability

The data described in the manuscript, code book, and analytic code will be made available upon request pending approval from the Gretchen Swanson Center for Nutrition personnel at (https://www.centerfornutrition.org/food-insecurity-measures/nutrition security).
