# Supplementary material for: Relationship between Food Security, Nutrition Security, and Diabetes: The Role of Supplemental Nutrition Assistance Program Participation
Source: Curr Dev Nutr. 2024 Mar 30;8(5):102153. doi: 10.1016/j.cdnut.2024.102153 (PMC11061707; doi:10.1016/j.cdnut.2024.102153)
Supplement: Multimedia component 1 [file mmc1.docx]

**Supplementary Figure 1. The Relationship between Nutrition and Food Security**

**
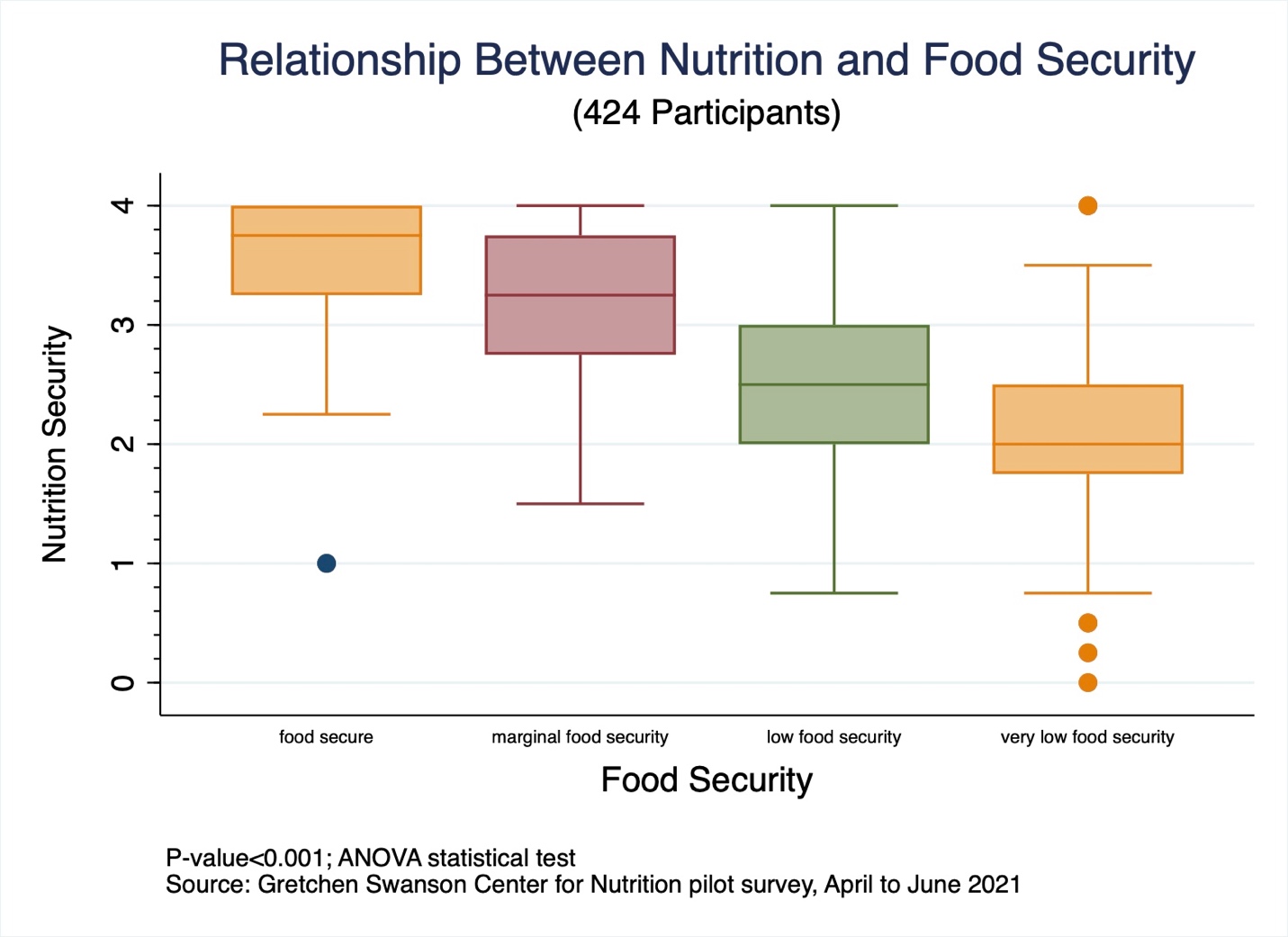
**

**Supplementary Figure 2. STROBE Participant Flow Chart**

Assessed for eligibility from April to June 2021

(n=517)

Missing data for diabetes (n=31), nutrition security (n=85), food security (n=42), gender (n=42), race/ethnicity (n=50), education (n=52), employment status (n=46), age (n=31), annual income (n=42), NSLP, WIC, SNAP, food pantry use (n=31), household with children (n=31), state (n=31), survey mode (n=31)

Included and Analyzed (n=441 participants) in Food Security models;

(n=399cparticipants) in Nutrition Security models

Abbreviations: *STROBE*, Strengthening the Reporting of Observational Studies in Epidemiology
